# Significant tricuspid regurgitation is associated with adverse outcomes in patients with transthyretin amyloid cardiomyopathy

**DOI:** 10.47487/apcyccv.v5i2.388

**Published:** 2024-06-24

**Authors:** Santiago Decotto, Juan María Iroulart, Guido Roveda, Eugenia Villanueva, María Adela Aguirre, María Lourdes Posadas-Martinez, Elsa Nucifora, Rodolfo Pizarro, Diego Pérez de Arenaza

**Affiliations:** 1 Cardiology Department, Hospital Italiano de Buenos Aires, Buenos Aires, Argentina. Cardiology Department Hospital Italiano de Buenos Aires Buenos Aires Argentina; 2 Internal Medicine Department. Hospital Italiano de Buenos Aires, Buenos Aires, Argentina; Internal Medicine Department Hospital Italiano de Buenos Aires Buenos Aires Argentina; 3 Instituto de Medicina Traslacional e Ingeniería Biomédica (IMTIB), Buenos Aires, Argentina. Instituto de Medicina Traslacional e Ingeniería Biomédica (IMTIB) Buenos Aires Argentina; 4 Hematology Department, Hospital Italiano de Buenos Aires, Buenos Aires, Argentina. Hematology Department Hospital Italiano de Buenos Aires Buenos Aires Argentina

**Keywords:** Amyloidosis, Heart Failure, Tricuspid Regurgitation, Amiloidosis, Insuficiencia Cardíaca, Insuficiencia Tricuspídea

## Abstract

**Objectives.:**

Patients diagnosed with transthyretin amyloid cardiomyopathy (ATTR-CM) often experience poor outcomes due to the development of heart failure (HF). Tricuspid regurgitation (TR) has been found to be correlated with adverse outcomes in patients with HF. This study aims to assess whether the presence of significant TR is associated to adverse cardiac outcomes in patients diagnosed with ATTR-CM.

**Materials and methods.:**

Retrospective study of ATTR-CM patients enrolled in the Institutional Registry of Amyloidosis (NCT01347047). Patients were categorized based on the presence of significant TR (moderate or severe according to current guidelines criteria) or absence of significant TR. All patients were followed up for 2 years to assess the incidence of the composite outcome of death or HF hospitalization.

**Results.:**

A total of 93 ATTR-CM patients were included. The mean age at diagnosis was 82.5 [IQR 75 - 86] years, 86% were male, and the mean left ventricular ejection fraction was 52% [IQR 43 - 60]. Among them, 32.3% (n = 30) patients had significant TR. Patients with significant TR had higher NTpro-BNP values (5308 vs 2454, pg/mL, p = 0.004), and a lower left ventricular ejection fraction (44 vs. 56%, p = 0.0002) compared to patients without significant TR. The incidence of the primary outcome was higher in patients with significant TR (77% vs. 30%, p<0.001). In a multivariate Cox regression analysis, only NTpro-BNP, as a numerical variable (HR 1.00, 95% CI 1.00005-1.0002, p = 0.001), and significant TR (HR 2.23, 95% CI 1.12-4.42, p=0.021) were independently associated with the composite outcome of death or HF hospitalization.

**Conclusions.:**

In patients diagnosed with ATTR-CM, the presence of significant TR was associated with worse outcomes.

## RESUMEN

**Objetivos.:**

Los pacientes con diagnóstico de miocardiopatía amiloidótica por transtiretina (ATTR) suelen presentar mal pronóstico, principalmente por el desarrollo de insuficiencia cardiaca (IC). Se ha descrito que la insuficiencia tricúspidea (IT) está relacionada con peor pronóstico en pacientes con IC. Este estudio tiene como objetivo evaluar el impacto pronóstico de la IT significativa en pacientes con diagnóstico de amiloidosis ATTR.

**Materiales y métodos.:**

Estudio de cohorte retrospectiva. Se incluyeron pacientes con diagnóstico de amiloidosis ATTR pertenecientes al registro institucional de amiloidosis de nuestra institución (NCT01347047). Los pacientes fueron divididos según la presencia o no de IT significativa (moderada o severa, de acuerdo a las guías de práctica clínica actuales). Se realizó un seguimiento con censura a los 2 años para evaluar la incidencia del punto final primario combinado de muerte u hospitalizaciones por IC.

**Resultados.:**

Se incluyeron 93 pacientes con diagnóstico de amiloidosis ATTR. La mediana de edad fue de 82,5 años [RIC 75-85], 86% fueron hombres. La mediana de fracción de eyección ventricular izquierda fue de 52% [RIC 43-60]. El 32,3% (n=30) de pacientes presentaron IT significativa al momento del diagnóstico de la amiloidosis ATTR. Aquellos con IT significativa tuvieron valores superiore de NT-proBNP (5308 vs. 2454, pg/mL, p=0.004) y menor mediana de fracción de eyección ventricular izquierda (44% vs. 56%, p=0,0002) comparado con los pacientes sin IT significativa. En el seguimiento a 2 años, la incidencia del punto final primario combinado fue mayor en pacientes con IT significativa (77% vs. 30%, p<0,001). En un modelo de regresión de Cox multivariada, solo el NT-proBNP (HR 1,00, 95% CI 1,00005-1,0002, p=0,001) y la IT significativa (HR 2.23, 95% CI 1.12-4.42, p=0.021) estuvieron asociadas de forma independiente con la ocurrencia del punto final primario combinado.

**Conclusiones.:**

En pacientes con diagnóstico de miocardiopatía amiloidótica por ATTR, la presencia de IT significativa se asoció a peor pronóstico en el seguimiento.

## Introduction

Transthyretin amyloidosis (ATTR) is a disease caused by abnormal fibrils derived from transthyretin, a protein primarily produced by the liver, which aggregate and deposit in tissues and organs ^1^^,^^2^. Cardiomyopathy is a common manifestation of ATTR amyloidosis, in which patients may develop symptoms of heart failure (HF) and experience a particularly poor life expectancy after diagnosis ^3^ ATTR cardiomyopathy (ATTR-CM) can be acquired through aggregation of wild-type TTR (ATTRwt) or inherited from a variety of genetic variants of TTR (ATTRv) ^4^.

The prognosis of patients with ATTR-CM is uncertain. The median survival from diagnosis in untreated ATTRwt patients is consistently approximately 3.5 years, but it depends on the stage of disease ^5^^,^^6^. Prognosis in the ATTRv population is even more uncertain, with variation in survival depending on genotype. Some series have reported a median survival in the range of 2.5 to 3.5 years in those diagnosed with HF ^7^.

Tricuspid regurgitation (TR), especially secondary or functional, is one of the most common valve diseases ^8^. TR greater than or equal to moderate is independently associated with worse survival in the general population ^9^^,^^10^. The presence of this heart valve disease has also been associated with a worse prognosis in patients with HF, both with reduced and preserved ejection fraction ^11^^,^^12^.

We have limited data available regarding the prognostic impact of significant TR in patients with ATTR-CM. The aim of this study is to compare the outcomes of patients with ATTR-CM, and significant TR and those without significant TR.

## Materials and Methods

### Study participants and design

This is a retrospective cohort study of adult patients (≥18 years of age), diagnosed with ATTR-CM (variant or wild type) at Hospital Italiano de Buenos Aires in Argentina, included in the Institutional Registry of Amyloidosis (IRA, clinicaltrials.gov/ct2/show/NCT01347047) from November 2007 to May 2021. We included patients who had a transthoracic Doppler echocardiogram performed within 6 months of the diagnosis of ATTR-CM. Patients diagnosed with TTRv without cardiac involvement and other forms of amyloidosis were excluded.

### Definitions and data sources

A confirmed diagnosis of ATTRwt was defined as myocardial uptake in pyrophosphate bone scintigraphy with a Perugini score greater than or equal to 2 and/or contralateral heart/chest ratio greater than or equal to 1.5 and/or diffuse myocardial uptake in SPECT imagines, or as a cardiac biopsy with positive Congo red staining in association with normal free light chains ratio. A confirmed diagnosis of ATTRv was defined as the identification of TTR gene mutation by complete gene sequencing associated with imaging tests that diagnose cardiac involvement (cardiac magnetic resonance, scintigraphy, or transthoracic echocardiogram).

Regarding electrocardiographic findings, microvoltage was defined as the amplitude of the QRS complexes in each of the six limb leads being equal to or less than 0.5 mV or equal to or less than 1.0 mV in the precordial leads from V1 to V6. Additionally, pseudo infarction pattern was defined as the presence of a pathological Q wave (1/4 of the amplitude of the R wave) or a QS pattern in at least 2 contiguous leads in the absence of left bundle branch block, ischemic heart disease, or segmental wall motion abnormalities on imaging tests.

TR was defined as significant in those patients with a severity of regurgitation greater than or equal to moderate according to the semi-quantitative and quantitative criteria outlined in the Guidelines for the Management of Valvular Heart Disease ^13^^,^^14^^).^ The echocardiogram closest to the pathology diagnosis was used to evaluate the presence of TR and its severity.

Hospital Italiano de Buenos Aires has been collecting amyloidosis cases on an ongoing basis in an ambispective registry, known as the IRA registry, since 2010. The registry includes baseline demographics, clinical history, comorbidities, physical examination, laboratory testing, and imaging data. Patients were followed up for overall survival, HF hospitalization, and other cardiac events. The frequency of follow-up was at the discretion of physicians. The information was collected using a standardized electronic case report form.

### Outcomes measures

The primary outcome was a composite of death or HF hospitalization. The secondary outcome was death for any cause. All patients were followed for 2 years, and administrative censoring was implemented to mitigate the competing risk of death from other causes.

### Statistical analysis

Continuous variables were presented as median and interquartile range [IQR] and compared using the Mann-Whitney test. Categorical data were reported as numbers and percentages and were compared using the Chi-Square test or Fisher’s exact test as appropriate. The two-year event ratios with their 95% confidence interval (CI) were calculated for both groups. Kaplan-Meier curves for event-free survival were generated for the time-dependent composite endpoints and compared using the log-rank (Mantel-Cox) test. Cox proportional hazards multivariate regression analysis was performed using variables with clinical significance that demonstrated an association with the outcome of interest.

### Ethical considerations

The study protocol was approved by the Institutional Ethics Committee and conducted in accordance with the principles outlined in the Declaration of Helsinki by the World Medical Association, the Standards of Good Clinical Practice, and the current legal regulations governing human research in Argentina.

## Results

A total of 93 patients were eligible for the analysis. The median age at ATTR-CM diagnosis was 82.5 years [IQR 75 - 86], and 86% were male. Only 5 patients had ATTRv. Overall, 48.9% of the patients had a history of heart failure prior to the diagnosis of amyloidosis. The median left ventricular ejection fraction was 52% [IQR 43 - 60], and 25% of patients had a reduced ejection fraction (< 40%). Patients’ baseline characteristics, stratified by TR severity, are summarized in Table 1. 

**Table 1:** Clinical characteristics of ATTR-CM patients with and without significant tricuspid regurgitation.

	Global (n=93)	Non-significant TR (n=63)	Significant TR (n=30)	p value
Age, years [IQR]	82 [75 - 86]	81 [74 - 86]	83 [76 - 87]	0.408
Male, n (%)	80 (86)	54 (86)	26 (87)	0.901
Hypertension, n (%)	77 (83)	53 (84)	24 (80)	0.622
Dyslipidemia, n (%)	48 (52)	32 (52)	16 (53)	0.877
Diabetes Mellitus, n (%)	23 (25)	15 (24)	8 (27)	0.740
Chronic renal failure, n (%)				
eGFR 30-60 mL/min/1.73m^2^	40 (43)	23 (37)	17 (57)	0.003
eGFR <30 mL/min/1.73m^2^	7 (8)	2 (3)	5 (17)	
Carpal tunnel Sd, n (%)	29 (31)	24 (38)	5 (30)	0.446
Neuropathy, n (%)	21 (23)	14 (22)	7 (23)	0.341
Prior CAD, n (%)	27 (29)	17 (27)	10 (33)	0.528
Atrial fibrillation/flutter, n (%)	54 (58)	28 (44)	26 (87)	< 0.001
Prior stroke, n (%)	13 (14)	7 (11)	6 (20)	0.248
Prior pacemaker, n (%)	9 (10)	5 (8)	4 (13)	0.411
NT ProBNP, pg/ml, [IQR]	3087 [2138-6173]	2453 [1473-4837]	5038 [3628-7178]	0.004
hsTnT, pg/ml, [IQR]	54 [34-87]	51 [29-72]	68 [37-112]	0.021
Microvoltage, n (%)	33 (35)	22 (38)	11 (39)	0.904
Pseudoinfarction pattern, n (%)	45 (48)	33 (57)	12 (44)	0.284

ATTR- CM: Transthyretin amyloid cardiomyopathy; TR: tricuspid Regurgitation; Sd: Syndrome; eGFR: Estimated glomerular filtration rate; CAD: coronary artery disease. NT-ProBnp: N-terminal prohormone of brain natriuretic peptide; hsTnT: high sensitivity Troponin T.

Overall, 32.3% of patients had significant TR in the transthoracic Doppler echocardiogram closest to the ATTR-CM diagnosis. Significant TR was classified as functional in all patients. The median age was similar in both groups. Patients with significant TR had a higher prevalence of prior atrial fibrillation or flutter (87 vs. 44%; P<0.001) and higher levels of NT-ProBNP (5308 vs. 2453 pg/ml; p = 0.004) and troponin at presentation (68 vs. 51 pg/ml; p = 0.02) compared with patients without significant TR.

There were no differences in HF medications between groups, and only 7 patients received tafamidis, 2 in the group with significant TR and 5 in the group without significant TR. Regarding the electrocardiogram, a similar proportion of pseudo-infarction pattern and microvoltage was observed in both groups. Additionally, concerning echocardiography parameters, patients with significant TR had a lower left ventricular ejection fraction (44% [IQR 39 - 53] vs. 56% [IQR 49 - 63]; p = 0.0002) compared to patients without significant TR. There were no differences in ventricular diameters or septal thickness between groups. As expected, more patients had right ventricular involvement and higher pulmonary systolic pressure in patients with significant TR (Table 2).

**Table 2:** Transthoracic echocardiographic characteristics of patients with and without significant tricuspid regurgitation.

	Non-significant TR (n=63)	Significant TR (n=30)	p value
LVEF, %, [IQR]	56 [49 - 63]	44 [39 - 53]	0.0002
EDVD, mm, [IQR]	43 [40 - 48]	43 [40 - 48]	0.972
ESVD, mm [IQR]	28 [25 - 32]	30 [27 - 41]	0.096
Septal thickness, mm, [IQR]	18 [14 - 20]	18 [14 - 20]	0.933
Severe AS, n (%)	6 (9)	4 (13)	0.579
RV involvement, n (%)	23 (38)	26 (87)	< 0.001
TAPSE, mm [IQR] (n=69)	19 [17 - 22]	14 [12 - 16]	< 0.001
SPP, mmHg [IQR] (n=71)	38 [29 - 46]	49 [42 - 59]	< 0.001

TR: Tricuspid Regurgitation; LVEF: left ventricular ejection fraction; EDVD: end diastolic ventricular diameter; ESVD: end systolic ventricular diameter; AS: aortic stenosis; RV: right ventricle; TAPSE: tricuspid annular plane systolic excursion; SPP: systolic pulmonary pressure.

During the 2 years follow-up, 43 patients (46%) experienced the primary combined outcome of death or HF hospitalization. Among them, 23 patients (24%) died, and 38 patients (40%) were hospitalized for HF. In the univariate analysis, the incidence of the primary outcome was higher in patients with significant TR compared to patients without significant TR (77% [n=23] vs 30% [n = 17]; p<0.001). Other variables that showed a statistically significant association with the composite primary endpoint were age, chronic renal failure, atrial fibrillation, NT-ProBNP, troponin, right ventricular involvement, the tricuspid annular plane systolic excursion (TAPSE) value, and pulmonary systolic pressure (Table 3). The left ventricular ejection fraction was not associated with the development of the primary outcome.

**Table 3:** Patient characteristics according to the occurrence or non-occurrence of the primary composite outcome (death or heart failure) at 2 years follow-up.

	Death or HF (n=42)	No Death or HF (n=51)	p value
Age, years [IQR]	84 [78 - 87]	80 [74 - 85]	0.047
Male, n (%)	37 (46)	44 (54)	0.975
Chronic renal failure, n (%)	29 (57)	18 (43)	0.003
Prior Atrial Fibrillation, n (%)	32 (59)	22 (40)	0.001
NT ProBNP, pg/ml, [IQR]	4895 [2659 - 8380]	2448 [1339 - 4607]	0.0007
hsTnT, pg/ml, [IQR]	82 [46 - 112]	36 [29 - 58]	0.0004
LVEF, %, [IQR]	49 [42 - 60]	55 [45 - 63]	0.106
RV involvement, n (%)	29 (59)	20 (41)	0.003
TAPSE, mm [IQR] (n=69)	16 [14 - 19]	20 [17 - 23]	0.005
SPP, mmHg [IQR] (n=71)	45 [40 - 54]	40 [29 - 49]	0.043

HF: heart failure; NT-ProBnp: N-terminal prohormone of brain natriuretic peptide; hsTNT: high sensitivity Troponin T; LVEF: left ventricular ejection fraction; RV: Right ventricle; SPP: systolic pulmonary pressure.

**Figure 1:**
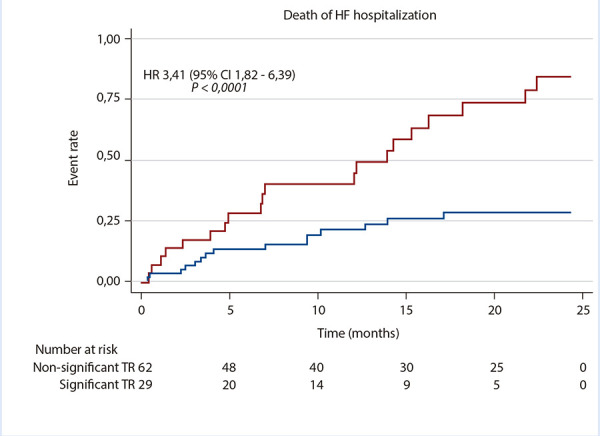
Kaplan-Meier curves for the composite primary outcome (death or heart failure hospitalization).

**Figure 2:**
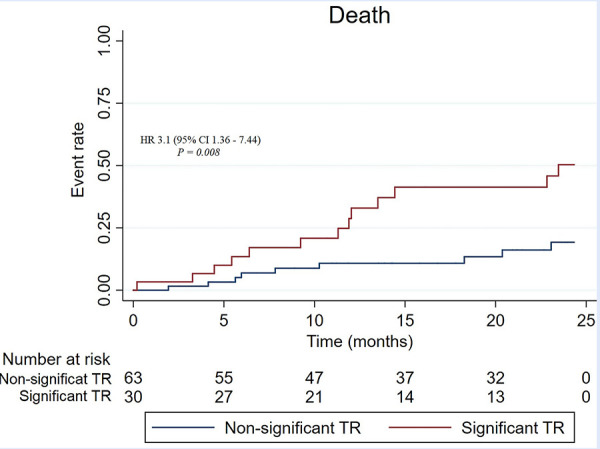
Kaplan-Meier curves for the secondary outcome (death).

Kaplan-Meier curves for the composite primary outcome and the secondary outcome are shown in Figure 1 and Figure 2. The two-year event ratio between patients with and without significant TR is shown in Table 4. Finally, in a Cox regression multivariate analysis that included age, sex, NT-pro-BNP, and significant TR, only NT-pro-BNP as a continuous variable (HR 1.00, 95% CI 1.00005 - 1.0002, p = 0.001) and significant TR (HR 2.23, 95% CI 1.12 - 4.42, p = 0.021) were independently associated with the composite endpoint of death or HF hospitalization.

**Table 4:** Two-year event rates between patients with and without significant TR.

	Non-significant TR (n=63)	Significant TR (n=30)	p value
Death or HF hospitalization rate [%], 95% CI	30 [19 - 43]	77 [58 - 90]	<0.001
Death, rate [%], 95% CI	14 [6 - 25]	43 (25 - 63]	0.002
HF hospitalization, rate [%], 95% CI	25 [15 - 38]	70 [50 - 85]	<0.001

TR: Tricuspid Regurgitation; HF: Heart Failure; CI: confidence interval

## Discussion

In our retrospective cohort, significant TR was associated with worse outcomes in patients with ATTR-CM, and this association was independent of age, sex, and NT-ProBNP value. These results underscore the significance of properly assessing this valvulopathy in patients diagnosed with ATTR-CM.

There is only a limited amount information available on this topic. Recently, Jerome Fagot *et al.* published a cohort study where they evaluated the prognostic impact of significant TR in this population ^15^. They included patients with light-chain amyloidosis (AL) and ATTR amyloidosis. Interestingly, the prevalence of significant TR was nearly identical (28%) in our cohort. Additionally, the clinical and echocardiographic variables of the patients were similar to ours. However, in the multivariate Cox analysis, moderate‐to‐severe TR was significantly associated with mortality only in patients with ATTR-CM but not in those with AL amyloidosis.

Regarding the characteristics of patients with significant TR in our study, they exhibited a higher prevalence of atrial fibrillation and right ventricular involvement, along with higher systolic pulmonary pressures, compared to patients without significant TR. These characteristics suggest that most cases were secondary TR. Pathophysiologically, this group of patients may have various causes of secondary TR, as they present with biventricular involvement and also because of the high incidence of atrial fibrillation, which leads to both left and right atrial enlargement ^16^. However, these described characteristics raise questions about whether significant TR acts as a risk factor in this population or simply as a marker of more advanced disease, and consequently, worse outcomes.

Another point to highlight is that in our cohort, the left ventricle ejection fraction was not associated with a higher risk of events. This finding is inconsistent with observations from other studies.^6^ The reason may be related to the limited power of the study. However, typically, other biomarkers are used to determine prognosis with greater certainty in ATTR-CM patients, such as NT-ProBNP levels. This biomarker has shown a strong association with events in multiple studies ^6^^,^^17^^,^^18^^)^ and is part of the main risk stratification systems in this pathology ^6^^,^^19^. In our cohort, NT-ProBNP was also independently associated with the primary outcome.

Finally, regarding the events rate, we observed a 24% mortality and 40% rate of HF hospitalization at 2 years follow-up. The survival rate is consistent with other studies assessing the prognosis of these patients, with an estimated 2-year mortality of 20 to 25% ^6^^,^^18^^,^^20^. As for the rate of HF hospitalization, when considering individuals who die, some studies have reported up to 60% of hospitalization at 2 years ^18^. This high event rate underscore the needs for interventions in this pathology. While further research is warranted in this area, significant TR could potentially become a therapeutic target in ATTR-CM patients in the future, especially given the recent advancements in percutaneous techniques. Although the development of transcatheter strategies for TR is still in the early stages, there is growing evidence to support the application of various approaches, including edge-to-edge repair and annuloplasty, to address unmet needs ^21^^-^^24^.

Our study has several limitations. Firstly, it shares all the limitations and biases associated with a retrospective, single‐site study. Additionally, the low number of patients and events did not allow us to perform multivariate models simultaneously adjusting for other clinically relevant variables. Furthermore, we did not have quantitative data for all patients with TR, as well as no measurement of the tricuspid annulus, both of which have demonstrated prognostic impact. Finally, as previously mentioned, left ventricle ejection fraction was not associated with the outcome of interest, likely due to the study´s lack of statistical power.

In conclusion, this retrospective study suggests that patients with ATTR-CM and significant TR experience worse outcomes compared to those without significant TR. These findings highlight the importance of further research and the potential inclusion of significant TR in risk scores for this population to better stratify the risk of events.
